# Flavins Act as a Critical Liaison Between Metabolic Homeostasis and Oxidative Stress in the Retina

**DOI:** 10.3389/fcell.2020.00861

**Published:** 2020-08-27

**Authors:** Tirthankar Sinha, Muna I. Naash, Muayyad R. Al-Ubaidi

**Affiliations:** Department of Biomedical Engineering, University of Houston, Houston, TX, United States

**Keywords:** riboflavin, retina metabolism, oxidative stress, redox potential, mitochondria, fatty acid oxidation, retbindin

## Abstract

Derivatives of the vitamin riboflavin, FAD and FMN, are essential cofactors in a multitude of bio-energetic reactions, indispensable for lipid metabolism and also are requisites in mitigating oxidative stress. Given that a balance between all these processes contributes to the maintenance of retinal homeostasis, effective regulation of riboflavin levels in the retina is paramount. However, various genetic and dietary factors have brought to fore pathological conditions that co-occur with a suboptimal level of flavins in the retina. Our focus in this review is to, comprehensively summarize all the possible metabolic and oxidative reactions which have been implicated in various retinal pathologies and to highlight the contribution flavins may have played in these. Recent research has found a sensitive method of measuring flavins in both diseased and healthy retina, presence of a novel flavin binding protein exclusively expressed in the retina, and the presence of flavin specific transporters in both the inner and outer blood-retina barriers. In light of these exciting findings, it is even more imperative to shift our focus on how the retina regulates its flavin homeostasis and what happens when this is disrupted.

## Introduction

The water-soluble vitamin, riboflavin (vitamin B2, aka lactochrome) was first isolated in 1879 from milk whey and purified as orange-yellow crystals. Subsequently, since Eijkman’s Nobel Prize-winning work in 1929, vitamins and their biological implications have become a matter of great interest to both biochemists and clinicians alike. As a vitamin, riboflavin is especially essential for human health due to its vast involvement in the bioenergetics, metabolism, growth, and survival of all cells (Powers et al., 2012; Ashoori and Saedisomeolia, 2014; Barile et al., 2016; Saedisomeolia and Ashoori, 2018; Suwannasom et al., 2020). So, the association between low riboflavin levels and various neurodegenerative disorders, metabolic dysfunctions, diabetes mellitus and inborn errors of metabolism, like multiple acyl-CoA dehydrogenation deficiency (MADD) (Reddi, 1986; Barile et al., 2016; Marashly and Bohlega, 2017; Xin et al., 2017; Saedisomeolia and Ashoori, 2018; Chen et al., 2019, 2020) is least surprising.

**FIGURE 1:**
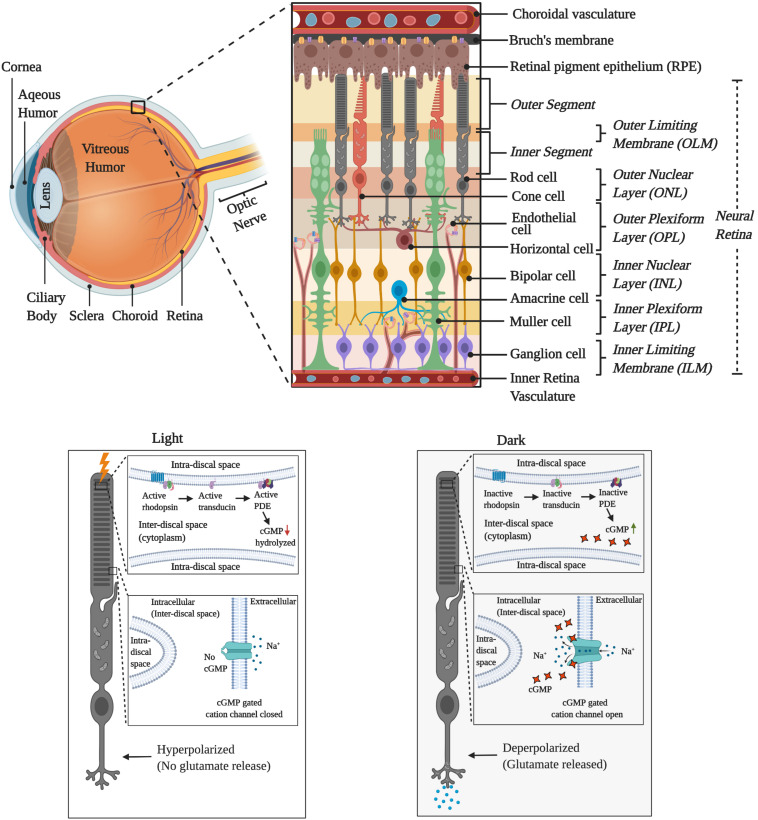
Graphical illustration of the cellular layers of retina and the activity during the light and dark cycles.

All proteins associating with flavins are collectively known as the flavoproteome (Lienhart et al., 2013). Being such a vast and diverse set of proteins, the extent of the flavoproteome involvement in numerous metabolic pathologies is only now surfacing out (Barile et al., 2016; Davis et al., 2016; Olsen et al., 2016; Fan et al., 2018; Balasubramaniam et al., 2019; O’Callaghan et al., 2019; Ryder et al., 2019). One such example is highlighted by Petrovski et al. (2015), where the authors describe the case of a 20-month-old female, who initially was being treated for a progressive neurological disease on the supposition that the illness was due to an autoimmune disease. But she was completely non-responsive to this treatment and only upon exome sequencing it was discovered that the child had a compound heterozygous genotype of two loss of function mutations in *SLC52A2*, a brain-specific riboflavin transporter. Following this discovery, she was immediately administered high-dose riboflavin therapy (10–70 mg/kg) and within 2–4 weeks of the treatment, most of her symptoms subsided (Petrovski et al., 2015). But recent discoveries are indicating that the effects of riboflavin deficiency are not limited to only neurological disorders in new-born babies (Olsen et al., 2016; Ryder et al., 2019). In another recent case report, a previously healthy 34-year old woman was suddenly presented with severe hearing and vision loss within 6 months and subsequently led to bilateral optic nerve atrophy, dysphagia, severe dyspnea, and quadriplegia (Camargos et al., 2018). Upon whole-exome sequencing, she was found to be carrying a novel homozygous insertion of 60 bp in *SLC52A3*, another riboflavin transporter (Camargos et al., 2018). High-dose riboflavin therapy (1,800 mg/day) for 6 months was able to improve her respiratory abilities and allowed her to walk with support but could not restore her neurosensory or visual loss (Camargos et al., 2018). Similarly, adult patients suffering from riboflavin deficiency due to malnutrition have been previously reported to have developed significant vision problems, including reduced rod and cone responses (Kruse et al., 1940). However, the majority of these studies date back a few decades and the renewed spotlight on vision loss due to riboflavin transporter mutation, calls for a comprehensive review of how diet or genetically induced riboflavin deficiency may affect the retina. The retina is a complex tissue lying at the back of the eye and formed of multiple layers of neuronal cells (Figure 1). Being metabolically active in both darkness and under light makes the retina (Figure 1) one of the most energy-consuming tissue as well as one of the most flavin enriched tissues. Thus, our focus in this review is to lay the foundation for future research on flavin homeostasis in the retina by highlighting the metabolic pathways flavins are intrinsically involved in and how dysregulation of these pathways is known to be associated with various debilitating retinal pathologies.

## Flavins and Their Unique Chemistry

**FIGURE 2:**
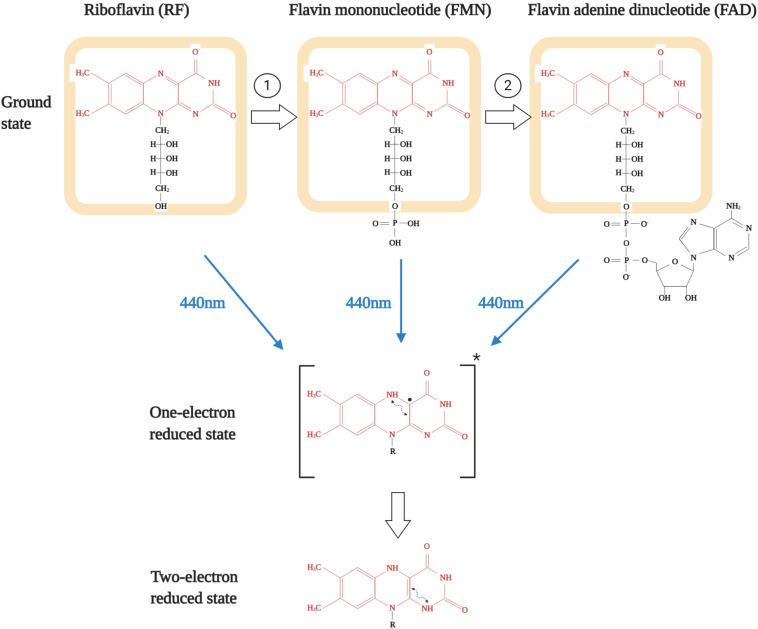
Structures of various flavins and their subsequent excitation upon blue-light exposure. Isoalloxazine ring is shown in red while riboflavin structure is shown as highlighted in orange. Reaction 1 is catalyzed by riboflavin kinase and reaction 2 is catalyzed by FAD synthetase.

The chemical structure of the tricyclic molecule riboflavin (aka 7,8-dimethylbenzo-pteridine-2,4-dione) is constituted of a ribitil side chain attached to an isoalloxazine ring, which is a benzene ring attached to a pteridine ring system (Figure 2; Massey, 2000; Powers, 2003). The presence of the pteridine ring gives it the name benzopteridine and reflects its relationship with another pteridine-based biochemical, i.e., folic acid (Thakur et al., 2017). For biological functioning, riboflavin is converted either to a phosphorylated (flavin mononucleotide, FMN) or an adenylated (flavin adenine dinucleotide, FAD) form of active redox coenzymes (Saedisomeolia and Ashoori, 2018). The conversion of riboflavin to FMN is catalyzed by the enzyme flavokinase or riboflavin kinase, which is an ATP dependent phosphotransferase (EC 2.7.1.26) (Reaction 1, as shown in Figure 2). Most FMN is then converted to FAD by FAD synthetase, which is an adenylyltransferase (EC 2.7.7.2) (Reaction 2, as shown in Figure 2; Muller, 1987; Powers, 2003). FMN is generated upon phosphorylation at the 5′-hydroxymethyl terminus of the ribityl side chain and that is converted to FAD upon addition of an adenylate group via pyrophosphate linkage (Shils and Shike, 2006). Even though multiple mutations in FAD synthetase have been reported to result in critical flavin deficient conditions, interestingly, none of these patients were found to have any structural or functional abnormalities in vision.

The importance of flavins is underlined by the chemistry of the compound (Massey, 2000). The biological activity of flavins is governed by the chemical versatility of the isoalloxazine ring. This is because it can exist in three different forms: oxidized, one-electron reduced, and the two-electron reduced state (Figure 2; Rivlin, 1970). It is important to note that these possible active states of all three flavins have mostly been detected in biological systems as protein-bound form and not in free form. This is of relevance as, compared to the free form in aqueous state, association with proteins markedly alters the stability of the one-electron reduced state (Joosten and van Berkel, 2007; McDonald et al., 2011). Though both non-covalent and covalent association can modulate flavin redox properties, but they act in a differential manner and is contingent upon the type of interaction (Massey, 1995; McDonald et al., 2011). Interestingly, most of the flavoenzymes have non-covalently than covalently bound FAD or FMN as cofactors (Joosten and van Berkel, 2007). But to further underline the importance of flavins in cellular respiration or metabolic biology, it is imperative to state that these are the foremost primary electron acceptors from soluble metabolites along with the nicotinamide coenzymes (NAD^+^/NADH). Being tricyclic gives flavins the ability to efficiently function as a transformer between electron donors and electron acceptors while the central dihydropyrazine ring of dihydroflavins is highly reactive to molecular oxygen, thus acting as a cofactor for reduction of molecular oxygen to hydrogen peroxide and also for reductive activity of monooxygenation reactions (Giulian et al., 1989).

## Flavins as Photosensitizers in the Retina

All three forms of flavins, i.e., riboflavin, FAD, and FMN, can act as photosensitizers (Oster et al., 1962). There are primarily two forms of photosensitization reactions they can take part in: (I) direct reaction between biomolecules and photosensitized flavins and (II) oxygen-dependent interaction between photosensitized flavins and biomolecules (Insinska-Rak and Sikorski, 2014; Fuentes-Lemus and Lopez-Alarcon, 2020; Fuentes-Lemus et al., 2020). However, both these oxidation reactions are undertaken when the isoalloxazine ring of flavins is excited upon exposure to blue light (Cardoso et al., 2012). As stated in the above section, both the one-electron and two-electron reduced forms of flavins (Figure 2) are highly reactive and thus leads to the formation of free radicals (Lopez-Alarcon et al., 2014). Due to this, flavins have been found to execute photosensitized oxidation of both lipid and protein biomolecules (Cardoso et al., 2013; Fuentes-Lemus and Lopez-Alarcon, 2020).

In type-I reactions, the two-electron reduced flavin (Figure 2) is quenched by the amino acid or lipid moieties most prone to be oxidized, resulting in the formation of a biomolecule radical cation and a flavin radical anion. The flavin radical anion can either react with oxygen to yield superoxide radical or accept a proton from the biomolecule radical cation or other donors to yield neutral free radicals. Subsequently, these neutral free radicals react with oxygen to form peroxyl radicals and eventually lead to the formation of hydroperoxides (Insinska-Rak and Sikorski, 2014; Fuentes-Lemus and Lopez-Alarcon, 2020). These species are further prone to decomposition in presence of redox active metal ions to yield alkoxyl radicals, which can add to the oxidative damage (Cardoso et al., 2012). Detailed investigations have revealed that among all the amino acids, tryptophan is most susceptible to such flavin-sensitized photo-oxidation in an oxygen-independent mechanism (Bhatia et al., 1991; Silva et al., 1991, 1994; Garcia and Silva, 1997; Fuentes-Lemus and Lopez-Alarcon, 2020). Excited state riboflavin binds to tryptophan under a light-induced reaction and leads to cytotoxicities like axonal degeneration and further cascade of photo-adduct formation (Silva et al., 1991; Lucius et al., 1998; Lopez-Alarcon et al., 2014). Lipid peroxidation is also a common pathological marker of blue light mediated photo-toxicity in the retina (Wenzel et al., 2005). The retina is known to harbor a hypoxic environment and is frequently exposed to blue light for an extended period of time even in artificially lit conditions, thus making a hotspot for such oxygen independent flavin photosensitization reactions (Jaadane et al., 2015, 2020; Shang et al., 2017). In type-II reactions, the two-electron reduced flavin directly reacts with O_2_ to convert it into the singlet state oxygen. This highly reactive form of oxygen can diffuse across a radius of 50–100 nm away from the site of formation and rapidly oxidize biomolecules like tryptophan, tyrosine, histidine, methionine, and cysteine amino acid residues since their kinetic rate constants are in the range of 10^6^–10^7^ M^–1^ s^–1^. This is interesting since elevated methionine and cysteine oxidation and multiple protein oxidation markers are a common phenomenon in various age related retinal pathologies, especially age-related macular degeneration (Organisciak et al., 1998; Marc et al., 2008).

Since the eye is directly exposed to light, the cytotoxic effect of flavins acting as photosensitizers is even more common and specifically such photo-induced protein oxidation of retinal ganglion cells have been found to compromise mitochondrial efficiency (Silva et al., 1991; Lucius et al., 1998; Osborne et al., 2014). Such protein oxidation and lipid peroxidation can compromise protein function, enzymatic activity, and membrane integrity, as well as elevate reactive oxygen species (Huvaere et al., 2010; Remucal and McNeill, 2011; Lopez-Alarcon et al., 2014; Fuentes-Lemus and Lopez-Alarcon, 2020). Also increased fluorescence of the oxidized form of mitochondrial flavoproteins has come up as a new tool to diagnose oxidative stress in retinal diseases, especially diabetic retinopathy and age-related macular degeneration (Spaide and Klancnik, 2005; Elner et al., 2008; Field et al., 2008, 2009; Litts et al., 2017; Andrade Romo et al., 2018). However, whether flavins as photosensitizers can affect the structure and function of photoreceptors or the retinal pigment epithelium (RPE) needs to be investigated. This is especially important given these two cells have the highest demand for riboflavin in the retina (Sinha et al., 2018). Blue light induced damage to the retina has been extensively investigated for decades and it is well established that the mechanism involves mitochondrial complexes as potential initiators of this phototoxic effect [reviews in Wenzel et al. (2005), Tao et al. (2019)]. Further mechanistic evaluation of blue light toxicity to the retina is outside the scope of this review. Here we focused on elucidating that flavins as photosensitizers can also be a major factor in blue light induced retinal damage and need to be considered in future mechanistic studies. Besides facilitating oxidation, exposure to light in acellular aqueous phase can lead to degradation of riboflavin itself, as has been previously reviewed (Sheraz et al., 2014). However, when we recently looked at the various conditions affecting riboflavin stability in the retina, we found protecting the retina from light (by dark adaptation) did not result in a change in retinal flavin levels (Sinha et al., 2018). Thus, it is likely that retinal riboflavin, similar to retinoids (Gonzalez-Fernandez et al., 2015), is somehow protected from degradation even though under constant exposure to light.

## Retinal Flavin Homeostasis and Oxidative Balance

The neural retina (NR) and the RPE together comprise the retina, which is one of the hotspots of highly reactive species in the whole body. The extremely high metabolic state of this tissue coupled with the high rate of oxygen consumption and the presence of multiple highly reactive phototransduction intermediates makes the retina vulnerable to various oxidative reactions. Thus, it is not surprising that to maintain homeostasis, the retina has developed an efficient system that counts on the ready availability of multiple electron acceptors and free radical scavengers. This is probably one of the reasons why both FAD and FMN are so highly enriched in the NR and the RPE (Sinha et al., 2018). The whole eye as an organ harbors arguably the highest level of riboflavin in the whole body (normalized to total protein content), and even though the cornea takes the major share of this, but it uses riboflavin mostly for structural purposes (Batey and Eckhert, 1990, 1991; Batey et al., 1992). The RPE and closely followed by the NR have the highest concentration of both the functional forms of riboflavin, FAD and FMN, and they are used critically as metabolic cofactors and free radical scavengers (Sinha et al., 2018, 2020b). It has been well elucidated that the glutathione based free radical scavenging system is highly dependent on flavins (Beutler, 1969). Glutathione peroxidase (GPx) reduces the intracellular H_2_O_2_ and toxic fatty acid hydroperoxides to water and in turn, GSH (reduced form) to GSSG (oxidized form). Glutathione reductase (GR), the enzyme that restores intracellular GSH (reduced form) levels by reducing GSSG (oxidized form) in an NADPH-mediated reaction, utilizes FAD as a cofactor (Higashi et al., 1978). Imbalance in the glutathione system has been shown to cause elevated retinal lipid peroxidation (Puertas et al., 1993). GSH depletion itself is a major cause of RPE ferroptosis and autophagy in a mitochondria independent manner (Sun et al., 2018). Absence of GSH downregulated RPE GPx (GPx4), a ferroptosis modulator, and increased LC3 expression, an autophagic marker (Sun et al., 2018). That riboflavin deficiency in experimental animals results in downregulation of GSH expression, reduced activity of GPx and increased lipid peroxidation in the eye, further raises the question if similar comorbidity happens in patients with retinal pathologies (Hirano et al., 1983; Horiuchi et al., 1984; Bates, 1991). If GSH cannot be recycled from GSSG due to reduced flavins, the RPE is unable to take up GSH exogenously and instead resorts to synthesizing it from secondary sources like glutamate, glycine, and cysteine (Davidson et al., 1994; Lu, 2013). This has a cascade effect on cellular metabolism as multiple metabolic resources now need to be repurposed to facilitate the adequate supply of these three amino acids. As an example, glucose is partially shunted away from glycolysis into the serine biosynthesis pathway, which is then converted to glycine (Sekhar et al., 2011; Lu, 2013; Panieri and Santoro, 2016; Sinha et al., 2020a, b). The RPE already harbors an efficient serine biosynthesis pathway in physiological conditions that may be upregulated to support enhanced GSH requirements. Interestingly, in the NR, even though the photoreceptors lack the repertoire for serine biosynthesis, Müller Cells (MC) has been shown to have the ability to synthesize serine and glycine for GSH production (Zhang et al., 2019).

Another close association between flavin deficiency and oxidative imbalance is via impaired mitochondrial redox balance, which is a major risk factor for ocular diseases like macular degeneration and diabetic retinopathy (Datta et al., 2017; Sinha et al., 2019). Patterson (Patterson and Bates, 1989) observed reduced oxygen consumption by the mitochondria in weanling rats fed riboflavin deficient diet associated with reduced weight gain per unit of food consumed (Tandler et al., 1969; Olpin and Bates, 1982; Patterson and Bates, 1989). Extremely hypoxic conditions can trigger reverse electron transfer and induce FMN to undergo reductive dissociation from complex-I of mitochondria, resulting in a robust decrease in complex-I function (Gostimskaya et al., 2007; Stepanova et al., 2017). Furthermore, significant accumulation of the reduced FMN can result in an equimolar amount of H_2_O_2_ in the mitochondrial matrix and can significantly contribute to oxidative stress (Massey, 1994; Kahl et al., 2018). Absence of flavins would also affect the β-oxidation of fatty acids in the RPE, which in turn would affect the flow of β-hydroxybutyrate to the retinal microenvironment, thus negatively impacting both the metabolic needs of the photoreceptors as well as the expression of oxidative stress resistance factors, as noted in other neurodegenerative disorders (Shimazu et al., 2013; Adijanto et al., 2014; Newman and Verdin, 2014). At the opposite extreme, Eckhert (Eckhert et al., 1993) reported that high levels of riboflavin can have harmful effects on the photoreceptors in a dose-dependent manner. He demonstrated a reduction in the number of photoreceptors in rats fed excess riboflavin (30 mg/kg) versus the recommended level (6 mg/kg). However, this is the only report exhibiting toxicity from excess riboflavin in the eye. Interestingly the following work by the same group showed that rats fed excess riboflavin were unable to increase the residual amount of flavins in the retina (Batey et al., 1992). So, what contributed to the degeneration is still a mystery. Indeed, it was shown that 10-fold higher levels of FMN can potently inhibit GR activity in *in vitro* conditions (Schorah and Messenger, 1975). But in physiological conditions, excess riboflavin is rapidly cleared out from the body (Yang and McCormick, 1967). Thus, to speculate that excess riboflavin could be responsible for oxidative damage, it is important to first investigate what conditions can result in a buildup of excess riboflavin in the retina.

## Role of Retinal Flavin Homeostasis and Glucose Metabolism in Vision

It has been shown that riboflavin plays a very prominent role in energy and glucose metabolism (Reddi et al., 1979). The retina is a metabolically active tissue with a high rate of energy demand and glucose consumption (Futterman and Kinoshita, 1959a, b). This is further validated by the highest activity of hexokinase in the inner segment of photoreceptors compared to the other cells of the NR as well as the brain (Burch et al., 1956; Lowry et al., 1961). This high activity is required for visual transduction as well as for the synthesis of new photoreceptor OS proteins, building new OS discs, and the shedding process. Using radioactive methionine, Young et al. showed that in rat, mouse, and frog, proteins synthesized in the photoreceptor IS are trafficked to OS in an ordered fashion, get accumulated in the lamellae in OS and subsequently are removed via shedding from the tip of OS in a light-dependent manner (Young, 1967). For this to effectively occur, a constant supply of energy and metabolites is required in the vicinity of the photoreceptors. To accommodate the high energy requirement, research on cattle and rabbit retinas demonstrated that high oxygen and glucose consumption occur via glycolysis, TCA cycle, and pentose phosphate pathway (Winkler, 1981).

Ames and colleagues showed that the retinal energy reserves are small, and withdrawal of glucose affects both the scotopic-a and b-wave of ERG (electroretinogram), which is an *in vivo* electrophysiological measurement of the retina (Ames et al., 1992). Surprisingly though, it had no immediate effect on oxygen consumption, indicating an alternate source of substrates for oxidative phosphorylation (Ames, 1992). This is in agreement with *ex vivo* results by Winkler (1981) showing that most of the glucose in the NR is converted to lactate and that inhibition of GAPDH (glycolytic enzyme) prevents the photoreceptors from having any extracellular potential, which is an *ex vivo* electrophysiological measurement of the photoreceptors and is similar to scotopic a-wave of the ERG. Oxygen withdrawal, on the other hand, leads to a Pasteur-effect with 2.7-fold increases in glycolysis and had a lower rate of decline in ATP production than in hypoxic conditions (Winkler, 1981; Ames et al., 1992). Thus, the authors demonstrated that retinal neurotransmission was heavily dependent on anaerobic glycolysis with it only contributing 18% of the total energy generated yet responsible for 80% of the total glucose consumed. Furthermore, they showed that phototransduction was dependent on oxidative metabolism with the dark current having the lion’s share of 41% of oxygen consumption. The large Pasteur-effect was explained by the hypothesis that in hypoxic conditions, dark current was partly supported by glycolysis.

Following published work describing the utilization of non-oxidative metabolism of glucose by neuronal cells of the retina, Pellerin and colleagues showed that upon glutamate release at excitatory synapses, glucose utilization and lactate production were stimulated (Pellerin and Magistretti, 1994). Thus, glycolytic lactate production in the retina is tied with neurotransmission in the dark current (Pellerin and Magistretti, 1994). Poitry-Yamate et al. also argued that this lactate was observed to be a better substrate for photoreceptor oxidative metabolism, even though they do take up both lactate and glucose (Poitry-Yamate et al., 1995). This was demonstrated by showing that about 70% of radioactive lactate released by the MCs was taken up solely by photoreceptors. Winkler et al. (2003) also used glucose to study whether MCs are the primary producers of lactate in rat NR, aerobically serving as the principal fuel for the photoreceptor mitochondrial functioning. Acknowledging species difference, the authors used rat NR as the avascular model and guinea pig NR as the vascular model. Interestingly, their results showed that under aerobic conditions, photoreceptors tend to depend upon glucose as the principal energy substrate, as long as the supply is adequate (Winkler et al., 2003). To specifically delineate the metabolism of the outer retina, Wang et al. highlighted the importance of oxidative phosphorylation and aerobic glycolysis-based lactate formation under light and darkness (Wang et al., 1997). The authors showed that glucose is the most efficient substrate, the preferred metabolite for the bulk of the energy production in the outer retina and that about 80% of this glucose utilized is converted to lactate in aerobic conditions (Wang et al., 1997). In physiological conditions, the dark cycle has greater oxygen consumption than during the light cycle (Wangsa-Wirawan and Linsenmeier, 2003), which suggests that if the oxygen demand/supply goes <1 unit then a hypoxic condition will arise in the retina. Linking oxygen consumption to the bioenergetics, Okawa et al. further looked at the difference in ATP consumption in light versus dark by rod photoreceptors (Okawa et al., 2008). The authors found that the vertebrate rods consume about 10^8^ ATP molecules per sec. The most dominant energy consumption is due to the ion fluxes associated with phototransduction and synaptic transmission. During daylight, the energy consumption drops by >75% due to perhaps inhibition of the dark response. The authors also showed that the cones are more energy consuming than rods (Okawa et al., 2008). Oxidative phosphorylation also seems to be the highest in photoreceptors compared to the rest of the NR since the highest cytochrome C activity (electron transport chain enzyme) is in the photoreceptors (Kageyama and Wong-Riley, 1984; Giulian et al., 1989) and the highest content of mitochondria resides in the photoreceptors (Cohen, 1961; Hoang et al., 2002). Stone et al. (2008) showed that mitochondrial localization in the avascular retinas of mouse, rat, and humans to be primarily in the IS but also a minor pool at the axon terminals while in the avascular retinas of wallaby, and guinea-pig to be only in the IS. Working on the avascular retinas of zebrafish, Linton et al. (2010) proposed that the major energy production in photoreceptors occurs in IS-mitochondria and that this metabolic energy, in the form of phosphocreatine, is transmitted to the synaptic terminal in darkness and toward the OS in light. However, in the vascularized retina, the dependency is less on creatine kinase (Linton et al., 2010). Perkins et al. (2003) estimated that in primates there are 10 times more mitochondria in cones than in rods, while in mice, cones have twice the mitochondria of rods. Using ferret, cat, and monkey, Riley et al. showed similar evidence demonstrating that the IS of cones is more densely packed with mitochondria than that of rods (Kageyama and Wong-Riley, 1984). This was supportive of the previous evidence that the cones consume more energy (Scarpelli and Craig, 1963).

The high density of mitochondria also reflects higher flavin requirement by the photoreceptors as most of the mitochondrial enzymes are flavin-dependent (Ragan and Garland, 1969). Furthermore, the above also supports the notion that the inner segment of a photoreceptor is fueled by flavin based oxidative phosphorylation while the functioning of the outer segment could be fueled by aerobic glycolysis. Ames found that the sodium-potassium ATPase transporters consumed about half of all the energy used by the NR, i.e., 49% of oxidative energy and 58% of glycolytic energy. It is important to bear in mind that the authors could not account for the fate of about 49% of the energy generated by oxidative metabolism (Ames et al., 1992). Since flavins play an important role in oxidative phosphorylation and all the critical components of oxidative phosphorylation are concentrated in the inner segment, it is logical to assume that the inner segment must have a pool of riboflavin derivatives. It has been shown that the activity of some enzymes involved in oxidative phosphorylation is significantly lower in riboflavin deficient rats (Zaman and Verwilghen, 1975), thus indicating how an imbalance in flavin homeostasis can affect the retinal energy metabolism.

Powers et al. showed in various cell culture systems the importance of riboflavin for energy generation (Lee et al., 2013). In fact, in absence of riboflavin, the cells seem to be under considerable oxidative stress due to the increasing supply-demand gap of ATP. Cells deficient in riboflavin have lower ATP levels and as flavokinase activity is less sensitive to ATP levels due to a 20-fold lower Km than FAD synthetase, the levels of FAD drop further with diminishing levels of ATP (Lee et al., 2013). So even if excess riboflavin is provided at this point, until ATP levels reach the threshold in a flavin-independent mechanism, riboflavin would not be converted to FAD and oxidative phosphorylation cannot begin again. Thus, it is essential to maintain riboflavin homeostasis in the NR, such that glucose metabolism keeps functioning efficiently to meet the energy requirement of the photoreceptors.

It is evident that oxidative phosphorylation and glycolysis for both ATP production and biomolecular substrate generation in the NR have very unique dynamics. We know how important flavins are for all these processes. Thus, it is justified that to maintain the dynamicity, effective flavin transport and homeostasis are crucial to the retina.

## Flavin Homeostasis and Lipid Metabolism in the Retina

Unlike other cells where lipids constitute 1% of their membranes, the photoreceptor cell membrane is constituted of 15% lipids (Scott et al., 1988). This highlights the significance of lipid metabolism to the proper functionality of photoreceptors. Riboflavin deficient chicken embryos exhibit dysfunctional fatty acid metabolism whereby the significantly reduced activity of FAD-dependent medium acyl CoA dehydrogenases leads to the build-up of C10, C12, and C14 fatty acids (Abrams et al., 1995). The authors argue that the impairment of fatty acid oxidation drains out the carbohydrate reserves and in turn negatively impacts energy metabolism. The authors note that the only difference between the chicken and the adult humans and rats under riboflavin deficiency is that there is an increase of dicarboxylic acids fin both adult mammals but not for the chicken embryo (Abrams et al., 1995). There are several reports in the literature showing an impairment of β-oxidation of fatty acids as an effect of flavin deficient diet and the rationale behind this could be the depressed activity of the flavin-dependent dehydrogenases (Olpin and Bates, 1982; Liao and Huang, 1987; Parsons and Dias, 1991). It is noteworthy that these dehydrogenases include all three alternate dehydrogenases; short, medium and long-chain fatty acyl-coenzyme A dehydrogenase. All of these dehydrogenases are involved in the very first step of β-oxidation of fatty acids (Tandler et al., 1969; Olpin and Bates, 1982; Patterson and Bates, 1989). Tandler’s work on isolated mitochondria from riboflavin deficient rat weanlings showed that the most drastic effect was on fatty acid oxidation, even though the oxidation of non-lipid substrates as succinate, pyruvate, glutamate, and α-ketoglutarate seemed to have a variable effect (Tandler et al., 1969). The rate-limiting step seemed to be the flavin-dependent acyl-CoA dehydrogenase activity (Tandler et al., 1969). The authors observed that the oxidation rates of both long-chain and intermediate chain fatty acid substrates dropped sharply as a result of ariboflavinosis (Tandler et al., 1969).

It is widely accepted that impaired β-oxidation of fatty acids can significantly contribute to vision loss and that it causes hypoglycemia (Taroni and Uziel, 1996; Kompare and Rizzo, 2008). Hypoketotic hypoglycemia, developed by patients having severely impaired β-oxidation of fatty acids (Taroni and Uziel, 1996) and 3-hydroxyacyl-CoA dehydrogenase deficiencies (Eaton et al., 2003; Tyni et al., 2004) have been reported to result in retinitis pigmentosa and peripheral neuropathy (Schrijver-Wieling et al., 1997; Tyni et al., 2004). Khan et al. (2011) showed that there is a close clinical effect of hypoglycemia on retinal function as detected by multifocal electroretinogram. This occurs in both normal subjects and those suffering from Type 1 diabetes, whereby, the central retina is preferentially affected (Khan et al., 2011). In another study, Adijanto et al. (2014) referred to a novel metabolic coupling between the RPE and the photoreceptors, by which, the photoreceptor outer segment membrane components get recycled back into ketones, to be fed into the oxidative phosphorylation of the photoreceptors (Adijanto et al., 2014). The authors show that RPE cells produce a high amount of β-hydroxybutyrate by β-oxidation of fatty acids, and it is then shuttled to the photoreceptors via the monocarboxylate transporter 1 (MCT1) (Adijanto et al., 2014), which is present in both the apical side of RPE and the photoreceptor IS (Philp et al., 2003). The substrate for ketogenesis via β-oxidation of fatty acids may come from the vast pool of fatty acids shed as photoreceptor OS, which is constitutively taken up by the RPE cells (Boesze-Battaglia and Schimmel, 1997). It is also possible that β-hydroxybutyrate, besides helping in the metabolic needs of the photoreceptors, may act as a neuroprotective agent by suppressing oxidative stress in the retinal microenvironment (Shimazu et al., 2013; Newman and Verdin, 2014). Thus, when the photoreceptor layer gets parched for riboflavin its fatty acid oxidation can be adversely affected. This, in turn, can have a cascading effect on the lipid metabolism of the RPE. Also, if riboflavin moves from the inner retina to the RPE (Kubo et al., 2017), then a similar condition of hypoglycemia can begin in the RPE further affecting the RPE functioning, leading to vision problems.

## Riboflavin Absorption and Transport

Since mammals have lost the ability to *de novo* synthesize riboflavin, it is acquired from the diet (Muller, 1987). Riboflavin absorption in the small intestine of rats and rabbits occurs across the brush border membrane in a specific carrier-mediated fashion, which is modulated by the level of riboflavin present in the vicinity (Said and Mohammadkhani, 1993; Subramanian et al., 2015). However, the body seems to get rid of excess plasma riboflavin within a span of a few hours, as has been reported for both animals (Yang and McCormick, 1967) and humans (Zempleni et al., 1996). In blood, riboflavin associates with plasma proteins like albumin (Wang et al., 2008) and reaches different parts of the body, enter various tissues either by diffusion or via specific transporters, and gets metabolically retained. In the last decade, it has been found that the brain has different transporters that are specific to riboflavin transport (Green et al., 2010; Haack et al., 2012; Johnson et al., 2012; Naik et al., 2014; Intoh et al., 2016). These are the same ones that have been identified earlier in other tissues. Recently, similar transporters were speculated to be present in the endothelial and epithelial cells of the inner and outer retina, respectively, as sh-RNA mediated knockdown and biochemical inhibition of these transporters resulted in decreased riboflavin uptake in TR-iBRB2, RPE-J and ARPE-19 cells (Said et al., 2005; Kubo et al., 2017, 2019). It was also shown that cultured RPE cells can take up riboflavin (Said et al., 2005), but this is yet to be validated *in vivo*.

**FIGURE 3:**
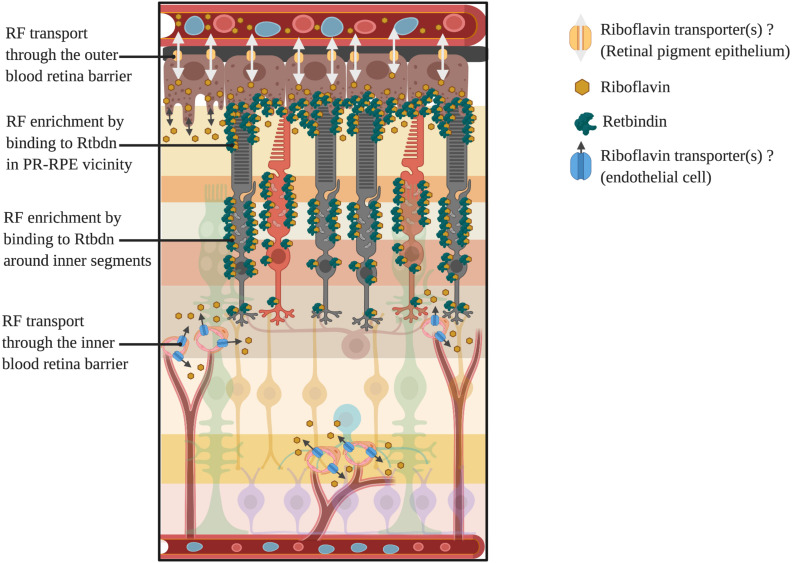
Potential routes of flavin transport into and out of the retina and enrichment around photoreceptors. Here we have shown the two potential routes for inflow and outflow of riboflavin through yet to be identified transporters present in both the RPE (outer blood retina barrier) and endothelial cells (inner blood retina barrier). Also shown is the localization of the retinal riboflavin binding protein, retbindin, and its enrichment of bound flavins around the photoreceptor inner segments and RPE-outer segment junction.

At this juncture, it is important to state that most of the cellular riboflavin is known to be phosphorylated as in metabolic trapping to prevent its diffusion out of the cell (Gastaldi et al., 2000). The free form of riboflavin diffuses out of the cells into the plasma and is eventually excreted out in the urine (Aw et al., 1983; Chastain and McCormick, 1987). However, it is not clear what happens to the riboflavin of the extracellular matrix. Extracellular proteins, like riboflavin carrier proteins, may bind to riboflavin and prevent it from diffusing back to the plasma. That may explain why riboflavin carrier proteins have been reported in all those tissues where the concentration of riboflavin is more than that of blood plasma, making these proteins as major players in flavin homeostasis in these tissues (Prasad et al., 1992; Bhat et al., 1995). Examples of these proteins are the riboflavin binding protein (RBP) of the chicken egg (Rhodes et al., 1959) and retbindin (Rtbdn) of the retina (Kelley et al., 2015). Based on these studies, a schematic depicting possible routes of flavin transport through the inner and outer retina is shown in Figure 3.

## Flavin Concentration in Different Tissues

The concentration of total bound and free flavins (riboflavin, FAD, and FMN) in each tissue is determined by the metabolic demands of the tissue (Muller, 1987). Hepatic and plasma levels have been quantified linking them to various pathologies (Patterson and Bates, 1989). Besides liver and plasma, analyses of flavin levels in the brain have recently gained importance due to riboflavin transporter diseases receiving attention (Yoshimatsu et al., 2016). But despite the higher metabolic activity of the retina (Ames et al., 1992), analysis of retinal flavin levels have received little attention. Euler and Adler were perhaps the first to report that the retina has a high riboflavin content (Pirie, 1943). Batey et al. then reported that rat NR harbors 46.5 ± 2.8 pmol/mg protein FAD, 17.6 ± 0.7 pmol/mg protein FMN, and 4.8 ± 0.34 pmol/mg protein riboflavin (Batey and Eckhert, 1990). Subsequently, riboflavin content in fish and mammalian eyes were found to be high compared to other tissues (Pirie, 1943). Later, Batey et al. (1992) further reported that the NR contained the highest FAD and FMN levels of all the ocular tissues in rabbits fed with three different concentrations of diet (Batey and Eckhert, 1991). It was also shown that increasing riboflavin intake 3 mg/kg animal weight did not increase total flavin content in the rat NR (Batey et al., 1992), thus suggesting a tissue requirement specific transport mechanism. The mammalian cell does not have the machinery to retain excess riboflavin and hence it is excreted out in the urine within a short time (Zempleni et al.). The riboflavin absorption, distribution, and clearance in rats have long been extensively studied by Bessey et al. using radioactive compounds (Bessey et al., 1958).

## The Flavoproteome of the Retina

The animal flavoproteome known so far can be widely divided into two types: One type is the coenzyme form of flavin derivatives binding to apoproteins either by covalent or noncovalent bonds (Macheroux et al., 2011; Leys and Scrutton, 2016). Examples of this type would be acyl-CoA dehydrogenase (Lienhart et al., 2013), succinate dehydrogenase (Lienhart et al., 2013), and glycerol-3-phosphate dehydrogenase (Lienhart et al., 2013) among several others. The other type is proteins that associate with flavins and mostly act as flavin carriers or function to enrich flavins in specific tissues (Powers et al., 2012). Examples of this type would be RBP found in a chicken egg (Rhodes et al., 1959; Ostrowski et al., 1968), riboflavin-carrier protein in pregnant rats (Muniyappa and Adiga, 1980a, b) and Rtbdn of the mammalian retina (Kelley et al., 2015).

In a comprehensive review, Lienhart provides a detailed report on the human flavoproteome (Lienhart et al., 2013). The author mentions that about 60% of the members of the human flavoproteome are associated with clinical pathologies (Lienhart et al., 2013). This underlines the importance of flavins in the proper physiological functioning of mammalian proteins. It is also important to note that most of the dysfunctionalities in flavoprotein pathologies are related to the mitochondrial, endoplasmic reticulum, and peroxisomal dysfunctionalities (Lienhart et al., 2013). This is not surprising in the case of the mitochondrial dysfunctionalities since a good number of the flavoproteins are located in the mitochondria and play a role in energy metabolism (Chance et al., 1967; Ragan and Garland, 1969). Flavoproteins associated endoplasmic and peroxisomal dysfunctionalities, on the other hand, point to the role flavins play in the exclusive functions performed by both organelles to aid in lipid metabolism (Lienhart et al., 2013).

Among all the flavoproteins, the mammalian retinal Rtbdn is unique. Rtbdn has the highest sequence homology to RBP of the chicken egg (Kelley et al., 2015). In mammals, primarily rod photoreceptors express Rtbdn and it is the only known riboflavin binding protein to be present in the retina (Kelley et al., 2015). What is most interesting is that Rtbdn is a peripheral membrane protein present on the extracellular side and attached to the membrane via electrostatic interactions (Kelley et al., 2015). Probably this enables the protein to bind to riboflavin present in the extracellular matrix. Rtbdn localizes mainly in two pools: one at the outer segment-RPE interface and the other around the inner segment of the photoreceptors (Kelley et al., 2015). Since multiple nutrients are exchanged between the NR and the RPE at the outer segment-RPE junction, it makes sense for Rtbdn to be highly enriched at this location to facilitate riboflavin transport back and forth between the NR and RPE (Figure 3). The other localization of Rtbdn is consistent with the fact that photoreceptors’ mitochondria are also present in highest concentration in the same region. Since flavins are essential for mitochondrial functioning and that photoreceptors’ mitochondria are highly active, it is possible that Rtbdn presence around the inner segment is chiefly to facilitate active flavin availability for oxidative phosphorylation. It would be worthwhile to validate this by investigating the rate of photoreceptor oxidative phosphorylation in absence of Rtbdn. But the importance of Rtbdn to a healthy retina is most obvious from the finding that in absence of Rtbdn, gradual degeneration is triggered (Kelley et al., 2017). Further, that this coincides with a decline in NR flavin levels, emphasizes how important Rtbdn is to maintain the retinal flavin demands. But mechanistic understanding behind this is lacking. Rtbdn may interact with other accessory membrane proteins which facilitate the internalization of flavins from Rtbdn itself. Also, since other flavoproteins are known to be unstable in absence of adequate flavins, whether the association of Rtbdn with the membrane is dependent on its binding to riboflavin is to be determined.

## Importance of Studying the Role of Riboflavin and Retbindin in the Retina

Blindness is reportedly the disease that can be caused by the most diverse set of gene mutations than any other disease known (Punzo et al., 2012). Mutations in over 300 different genes or gene loci are known to be associated with inherited retinal diseases (IRDs) (RetNet, 2020, Accessed May 27th, 2020). Metabolic vulnerability and predisposition to oxidative stress have been touted as an underlying facilitator for such multi-genic retinal diseases (Leveillard et al., 2019). Unsurprisingly, therapeutic interventions targeted to ameliorate metabolic stress has shown that it is indeed a promising approach to treat such a wide spectrum of blinding diseases (Hurley and Chao, 2020; Wert et al., 2020). Given the importance of flavins in many metabolic pathways that are essential for retinal homeostasis, it is imperative to maintain optimum levels of flavins for a healthy retina. As reported by Amemiya (2007), the retina of rats fed with riboflavin deficient diet for 3 months showed clear signs of degeneration with edematous and disoriented MCs, disintegrating OS discs and RPE full of an abnormal number of lamellae. Interestingly, these seemed to be reversible since animals recovered when they were placed on a riboflavin enriched diet. In absence of literature presenting ultrastructural images of the effects of long term riboflavin deficiency, one can assume that the high number of lamellae in the RPE even after 7 h (shedding stops usually within few hours after the onset of the light cycle), is due to either slower rate of phagocytosis by the RPE or enhanced degenerating OS contributing to extended phagocytosis. That the tip of the OS seems to be affected may support the line of thought that the OS/RPE interface is affected, creating a stressful environment for the interphotoreceptor matrix in a state of ariboflavinosis.

The absence of Rtbdn also leads to a reduction in NR’s flavin levels, which corresponds with retinal degeneration (Kelley et al., 2017). Most interesting is that even though rods specifically express Rtbdn, the cones are affected in Rtbdn’s absence (Kelley et al., 2017). This is further supportive of previous evidence that rods express specific proteins that are essential for cone health (Chalmel et al., 2007). Further, a significant reduction in retinal flavin levels in only rod specific degeneration models indicated they are responsible for the majority of retinal flavins (Sinha et al., 2018). However, the fact that there still existed some amount of flavins is indicative that there must be another [likely a photoreceptor independent (“?” in Figure 3)] mechanism, which may be essential for flavin homeostasis of the inner retina. Thus, rod death during retinitis pigmentosa or other retinal pathologies could result in a local ariboflavinic environment around the photoreceptors, leading to a starving condition for the cones, triggering cone death that usually follows rod death as observed in RP patients and in models of IRDs (Punzo et al., 2012). Due to its role in retinal homeostasis, when Rtbdn was eliminated from a model of cone-rod dystrophy, the degenerative process was exacerbated (Genc et al., 2020). Expression of elevated levels of Rtbdn during retinal degeneration further indicated that the protein could be playing a protective role (Genc et al., 2020). It is possible that when confronted with a stressful condition as degeneration, the retina needs a higher level of energy, hence an increased need for flavins, to mitigate this insult and thus overexpresses Rtbdn. It is worth mentioning that the absence of Rtbdn triggered a compromise in retinal vasculature integrity and led to the formation of vascular tufts (Genc et al., 2020). It would be worthwhile to see if such a trend is mimicked in other models of retinal degeneration as well and whether there is a difference between the behaviors of models of cone dominant mutations versus those resulting from rod dominant mutation. A previous study (Venkataswamy, 1967) described the various ways ariboflavinosis can affect different parts of the eye. The authors cite Davson’s (1949) chapter (Venkataswamy, 1967), highlighting the rich flavin content in the retina and its deficiency linked to night blindness. They also reported that patients suffering from ariboflavinosis showed resolution of their night blindness following a 10-days course of 10 mg riboflavin/day injections. The authors mention two previous reports by Pollak in 1945 and by Gordon in 1939, emphasizing the ability of riboflavin alone to improve dark adaptation (Pollak, 1945). However, supplementary evidence is lacking on these lines and needs to be validated by further research. Given the fact that pathology as riboflavin transporter disease improves with flavin-enriched diet (Timmerman and De Jonghe, 2014; Bashford et al., 2017), it is interesting to see if high riboflavin diet for an extended period can rescue RP animal models from photoreceptor degeneration.

## Future Perspective

Putting all the research into perspective, it seems very important to look at both: (1) the role of flavin homeostasis in retinal physiology as well as (2) the role of flavin homeostasis in retinal pathologies, especially those where metabolic vulnerability and oxidative stress susceptibility is involved. One of the tools available to us to assess the role of flavins in retinal homeostasis is the Rtbdn knockout model (Kelley et al., 2015). The presence of a highly regulated barrier like the blood-retinal barrier, combined with the high energy metabolism in the retina, such specialized proteins seems critical for retinal homeostasis. It is possible, that like RBP of the egg, Rtbdn, helps in the transport of riboflavin across the interphotoreceptor matrix and thus maintaining the high intracellular pool of riboflavin in the photoreceptors. It will also be beneficial to specifically identify the transporters that may be involved in riboflavin transport to the retina and investigate what happens if their levels are selectively altered, both in health and disease. Future research should also focus on identifying mutations in either Rtbdn or any of the riboflavin transporters that cause or modify retinal degenerative diseases. Moreover, since so little is known about any of retinal riboflavin carrier proteins, biochemical and biophysical characterization of both Rtbdn and riboflavin transporters would provide us with a greater understanding as to how such high flavin levels are maintained in the retina. Similarly, it will be worthwhile to investigate if flavin deficiency confounds retinal dystrophy in patients and whether maintaining optimum flavins provides better prognosis when the retina is under metabolic or oxidative stress. Thus, it seems important to do more work on the role of flavin homeostasis with respect to the structural and functional integrity of the retina and further our knowledge on the criticality of this underappreciated vitamin to the retina.

## Author Contributions

TS, MN, and MA-U contributed to writing and editing the manuscript. All authors contributed to the article and approved the submitted version.

## Conflict of Interest

The authors declare that the research was conducted in the absence of any commercial or financial relationships that could be construed as a potential conflict of interest.
